# Decoding the Substrate Supply to Human Neuronal Nitric Oxide Synthase

**DOI:** 10.1371/journal.pone.0067707

**Published:** 2013-07-09

**Authors:** Alexandra Simon, Susanne Karbach, Alice Habermeier, Ellen I. Closs

**Affiliations:** Department of Pharmacology, University Medical Centre of the Johannes Gutenberg University, Mainz, Germany; Albany Medical College, United States of America

## Abstract

Nitric oxide, produced by the neuronal nitric oxide synthase (nNOS) from L-arginine is an important second messenger molecule in the central nervous system: It influences the synthesis and release of neurotransmitters and plays an important role in long-term potentiation, long-term depression and neuroendocrine secretion. However, under certain pathological conditions such as Alzheimer’s or Parkinson’s disease, stroke and multiple sclerosis, excessive NO production can lead to tissue damage. It is thus desirable to control NO production in these situations. So far, little is known about the substrate supply to human nNOS as a determinant of its activity. Measuring bioactive NO via cGMP formation in reporter cells, we demonstrate here that nNOS in both, human A673 neuroepithelioma and TGW-nu-I neuroblastoma cells can be fast and efficiently nourished by extracellular arginine that enters the cells via membrane transporters (pool I that is freely exchangeable with the extracellular space). When this pool was depleted, NO synthesis was partially sustained by intracellular arginine sources not freely exchangeable with the extracellular space (pool II). Protein breakdown made up by far the largest part of pool II in both cell types. In contrast, citrulline to arginine conversion maintained NO synthesis only in TGW-nu-I neuroblastoma, but not A673 neuroepithelioma cells. Histidine mimicked the effect of protease inhibitors causing an almost complete nNOS inhibition in cells incubated additionally in lysine that depletes the exchangeable arginine pool. Our results identify new ways to modulate nNOS activity by modifying its substrate supply.

## Introduction

Nitric oxide (NO), produced by nitric oxide synthases (NOS) from the cationic amino acid arginine, is an important second messenger molecule involved in several physiological actions: Vascular NO produced by endothelial NOS (eNOS) relaxes smooth muscle cells and thus decreases blood pressure. In addition, it inhibits smooth muscle cell proliferation, platelet aggregation and leukocyte adhesion, thus acting as a vasoprotector [1]. In the immune system NO produced by the cytokine-inducible iNOS in large quantities destroys pathogens and microorganisms. It is however also involved in autoimmune processes [2] and tumor development [3]. NO produced by neuronal NOS (nNOS) is known to work as an important modulator of neuronal function acting on the release of neurotransmitters [4]. nNOS knockout mice show significantly reduced levels of tyrosin-hydroxylase and phenylethanolamine-N-methyltransferase (both important enzymes for catecholamine-production) if compared to wild type mice [5]. Furthermore, NO plays an important role in synaptogenesis, long-term potentiation and long-term depression [6] thus affecting synaptic plasticity and memory function [7]. An important influence of NO has also been reported on neuroendocrine secretion, in particular on the regulation of the endocrine stress response [5], [8]. However, also in the brain NO may have harmful effects: an excessive NO production, due to an increased expression or activity of nNOS, results in dysfunction of the mitochondrial electron transport chain, leading to cellular energy deficiency and neurotoxicity [9] [10]. Under certain pathophysiological conditions such as stroke, nNOS produces also superoxide that reacts rapidly with NO to peroxynitrite, a highly reactive oxidant [11]. Here, the excessive activity of the calcium-dependent nNOS is triggered by an unbraked presynaptic glutamate release after cerebral ischemia, followed by calcium entry via N-methy-d-aspartate-receptors (NMDAR) [12] [13]. The described neuronal nitro-oxidative stress has also been described to play a crucial role in the development of Alzheimer’s [14] as well as Parkinson’s disease [13] [7] [15]. It has recently been shown that stimulation of the neurotrophin receptor TrkB on astrocytes drives nitric oxide production and by this way triggers neurodegeneration [16]. Currently, working groups focus on the design of selective neuronal nitric oxide synthase inhibitors for the prevention and treatment of neurodegenerative diseases [7]. However, in the light of the beneficial effects of NO in the central nervous system mentioned above, a complete nNOS inhibition may also have detrimental effects.

Another approach to regulate nNOS activity could be via the supply of the substrate L-arginine to the enzyme. However to date, little is known about arginine sources of human nNOS. Therefore, a better understanding of the substrate supply to nNOS is necessary to find new possibilities to treat illnesses where an increased level of NO produced by nNOS causes damage in the brain (as Alzheimer’s or Parkinson’s disease, multiple sclerosis and stroke) [13] [17] [18]. Arginine can be taken up from the extracellular space by specialized transporters such as cationic amino acid transporters (CAT, isoform 1, 2A, 2B and 3) or system y^+^L transporters (4F2hc/y^+^LAT, isoforms 1 and 2) [19]. Both transport systems catalyze the exchange of cationic amino acids. In addition, the system y^+^L transporters mediate the exchange of intracellular cationic amino acids versus extracellular neutral amino acids and Na^+^ thus providing an active efflux rather than an influx pathway for arginine [19]. Apart from transport, arginine can be generated intracellularly by protein breakdown or conversion of citrulline to arginine by the successive action of argininosuccinate synthase and lyase. These so called recycling enzymes as well as several CAT isoforms are expressed in a distinct pattern in the rat brain [20]: CAT-1 has been described to be ubiquitously expressed in neuronal and glial cells, with highest expression in the brainstem, cerebellum and telencephalon. Argininosuccinate lyase also manifests a ubiquitous neuronal and glial expression in all parts of the rat brain, however at low levels. Argininosuccinate synthase localizes to neurons with highest expression in cerebellum and brainstem and in scattered cells of the midbrain and the telencephalon. It is however not detectable in glia cells. nNOS is restricted to scattered neurons in telencephalon, diencephalon, midbrain, brainstem and cerebellum [20]. Braissant et al found that all nNOS expressing cells investigated, also expressed both recycling enzymes [20].

Our previous work with endothelial cells revealed that both, arginine transport from the extracellular space and generation from intracellular sources, are important substrate sources for the endothelial NOS (see scheme in Figure S1): Arginine is easily transported into and out of cells by transport systems in the plasma membrane (hCATs, y^+^LATs). Besides, there are intracellular arginine sources that differ concerning their exchangeability with the extracellular space via exchange transporters as well as their accessibility for eNOS. Exposure of EA.hy926 endothelial cells to high concentrations of lysine does not lead to a downregulation of NO synthesis by eNOS, in spite of pronounced arginine extraction from the cells by arginine-lysine exchange via hCAT1 and y^+^LAT transport systems [21] [22]. We thus defined two different intracellular arginine pools: Pool I that can be depleted by extracellular lysine and pool II that is not freely exchangeable with the extracellular space, but accessible to eNOS [21] [22]. One part of pool II (referred to as pool IIA) consists of citrulline to arginine recycling. This part can be depleted by glutamine and histidine, both substrates of the system N transporter, most likely by removal of intracellular citrulline and hence prevention of arginine regeneration from its precursor (Figure S1). The second part of pool II (referred to as pool IIB) is replenished by protein breakdown. It is depleted by histidine, probably by exchanging intracellular peptides by extracellular histidine through the peptide histidine transporter PHT1.

In the present study, we asked if the different substrate pools detected for eNOS are also available for nNOS. The presence of membrane transporters as mentioned above indicates a functional arginine pool I in neurons. Bae et al. demonstrated arginine transport in murine tyrosine hydroxylase positive CAD cells to be regulated by changes in membrane potential and suggested that arginine supply in neuronal cells adapts “in a moment-to-moment fashion to the changing needs of the neurons” [23]. This is in line with the dependence of CAT activity on membrane potential [24] [25] [26]. It is however not known whether nNOS - like iNOS - relies completely on the exchangeable substrate pool I or if it has - like eNOS - also access to intracellular arginine sources (pool II). In the present study, we thus examined nNOS activity in human A673 neuroepithelioma and human TGW-nu-I neuroblastoma cells, answering the following questions:

1. Are there intracellular arginine sources, not freely exchangeable with the extracellular space, that can be used as substrate source for nNOS (pool II)?
2. Are neuronal cells capable of recycling citrulline to arginine and – if yes – can this arginine be used as nNOS substrate (pool IIA)?
3. Does protein degradation (lysosomal/proteasomal) play a role for substrate supply of nNOS (pool IIB)?

## Materials and Methods

### Cell Culture

The human neuroepithelioma cell line A673 [27] was obtained from the European Collection of Cell Cultures (ECACC, Salisbury, United Kingdom). The human neuroblastoma cell line, TGW-nu-I was kindly provided by Dr. Esumi [28] [29]. The human endothelial cell line EA.hy926 was a gift from Dr. Edgell [30]. The murine macrophage cell lines J774A.1 and the rat lung fibroblast cell line RFL-6 were obtained from ATCC, Bethesda, MD, USA.

A673 cells were cultivated in IMDM-medium with 10% fetal bovine serum (FBS). TGW-nu-I cells were grown in Earl’s MEM with 10% FBS**.** EA.hy926 and murine J774A.1 cells were grown in Dulbecco’s modified Eagle’s medium (DMEM), supplemented with 4 mmol/l glutamine and 10% FBS. RFL-6 cells were grown in F-12 medium, supplemented with 4 mmol/l glutamine and 15% FBS. All cells were kept at 37°C in 10% CO_2_. J774A.1 cells were stimulated with 1 µg/ml bacterial lipopolysaccharide (LPS) for 12h prior to NO measurements.

Cells were regularly tested for mycoplasma infection using 4′,6-diamidine-2′-phenylindole dihydrochloride (DAPI, Roche Molecular Biochemicals, Mannheim, Germany). No contamination was detected.

### RFL-6 Reporter Cell Assay

NO was measured indirectly by the induction of cGMP formation in RFL6 reporter cells [31]. Confluent NO-producing cells in 6- or 24-well plates were washed twice in Locke’s solution (LS; composition [mmol/l]: 154 NaCl; 5.6 KCl; 2 CaCl_2_; 1 MgCl_2_; 10 HEPES; 3.6 NaHCO_3_; 5.6 glucose), then preincubated where indicated in LS supplemented with either arginine or lysine (1 mmol/l) and for further 30 min in the same solution, respectively, supplemented additionally with 20 U/ml superoxide dismutase (Roche Molecular Biochemicals) and the indicated compounds at 37°C (two buffer changes each incubation). All amino acids applied were in the L-isoform. For the actual NO measurement, cell supernatants were renewed by the same solution, respectively, supplemented with 0.6 mmol/l 3-isobutyl-1-methylxanthine (Serva) and 10 µmol/l calcium ionophore A23187. After a 2 min incubation at 37°C, supernatants were transferred to the RFL-6 cells (that had been washed twice in LS and preincubated in LS supplemented with 0.6 mmol/l 3-isobutyl-1-methylxanthine for 30 minutes at 37°C). After a 2 min incubation at 37°C, supernatants were removed from the RFL-6 cells, 100 µl ice-cold sodium acetate buffer (20 mmol/l, pH 4.0) was added, and the cells were rapidly frozen in liquid nitrogen. For repeated measurements, the same NO-producing cells were incubated for 4 min with fresh LS containing the indicated supplements (one buffer change after 2 min). The cell supernatant was then transferred to new RFL-6 cells. The cGMP content of the RFL-6 cells was determined by radioimmunoassay as described previously [31] [32]. Values obtained were calculated as % of the mean of the values obtained from the corresponding control cells incubated in 1 mM arginine in experiments performed on the same day. The values obtained with different batches/passages of RFL-6 cells varied in a range between about 5 and 150 and 0.5 and 10 pmol cGMP per 10^6^ RFL-6 cells for A673 and TGW-nu, respectively, due to differences in the sensitivity of the reporter cells [33].

### Quantitative Reverse Transcription and Polymerase Chain Reaction (qRT/PCR)

Total RNA was isolated using the RNeasy Mini Kit (Qiagen, Hilden, Germany) and quantified by the absorption of the eluate at 260 nm. One-Step qRT/PCR was performed with the QuantiTect RT/PCR Kit (Qiagen, Hilden, Germany) in 25 µl reactions in a 96-well spectrofluorometric thermal cycler (iCycler, Bio-Rad, München, Germany) (dNTPs: 400 µmol/l each). For real-time PCR (MgCl_2_∶5.3 mmol/l, 94°C 15 s, 60°C 60 s), the following oligonucleotides served as sense and antisense primers, respectively: GATCAGCGAGATCTTTGCAAGTATC and CATTATGGCACTCTGCATGGAC (human PHT1), AGCCTCAAGATCATCAGCAATG and CACGATACCAAAGTTGTCATGGA (human GAPDH). Taqman hybridization probes were double labeled with 6-carboxyfluorescein (FAM) as the reporter fluorophore and carboxytetramethyl-rhodamine (TAMRA) as the quencher: 5′-6FAM-AGGCCTGGAATTTGCATACTCAGCTGCp-3′ (PHT1 probe) and 5′-6FAM-CTGCACCACCAACTGCTTAGCACCCXTp-3′ (GAPDH probe). Fluorescence was monitored at each 60°C annealing/extension step.

### Analysis of the Intracellular and Extracellular Arginine Content by High Performance Liquid Chromatography (HPLC)

Arginine was measured by high performance liquid chromatography (HPLC) using precolumn derivatization and fluorescence detection. Cells were washed 2 times in ice-cold phosphate buffered saline (PBS), lyzed in 0.5 ml ice-cold 70% EtOH (30 min, 4°C) supplemented with 1 nmol monomethyl L-arginine and centrifuged at 14,000 g for 10 min. Supernatants were applied to Oasis MCX ion exchange cartridges to separate basic amino acids. Eluates were dried by vacuum centrifugation and resuspended in 100 µL 0.5 mol/l borate buffer pH10. 50 µl sample was incubated with 12 µl OPA reagent (30 mg o-phthaldialdehyde, 2.7 ml methanol, 300 µl 0.5 mol/l borate buffer pH 10, 30 µl 2-mercaptoethanol), and 20 µl acidic acid (1 mol/l) for 180 sec. Amino acid derivatives (15 µl) were separated on a Nova-Pak column (C18, 4 µm 3.9×300 mm, Waters, Eschborn, Germany) using a gradient of 50 mM sodium acetate, pH 6.8 supplemented with 0.044% triethylamine and acetonitril (flow rate 1 ml/min). Fluorescence (excitation wavelength, 330 nm; emission wavelength, 450 nm) was monitored with a Bischoff 8470 fluorimeter and quantified using the analysis program McDAcq (Bischoff, Leonberg, Germany).

### Thin Layer Chromatography (TLC)

For analysis of the conversion of citrulline to arginine, confluent cells grown in six-well-plates were washed twice in LS and then preincubated for 2 h in 300 µl LS supplemented with lysine (1 mmol/l). Cells were then incubated with ^14^C-labelled citrulline (18.4 µM; specific activity 54.5 Ci/mol;  = 2016 GBq/mol) for one hour and the indicated amino acids. A conversion of arginine to citrulline was inhibited by the addition of 1 mM L-N^G^-nitro-arginine methyl ester (L-NAME).

Supernatants of 300 µl were used for TLC after having been dissolved in 1.5 ml 100% methanol. Cells were washed twice in LS, permeabilized in 100 µl LS and 500 µl methanol, centrifuged 10 min at 10,000 g at 4°C, vacuum-dried and finally re-dissolved in 6 µl of 50% methanol. 5 µl of each sample were then transferred with capillary pipettes into separate spots of the TLC plate coated with silica gel. The samples were then separated over a distance of about 16 cm with 50 ml of solvent (composed of chloroform : methanol : ammoniumhydroxid : water in the ratio: 0.5∶ 4.5∶ 2.0∶ 1.0). After drying, the radioactivity of individual spots on the TLC plate was determined using a phosphorimager (Biorad) and analyzed with the programm „Multi Analyst“.

### Statistical Analyses

Statistical analysis was performed using either unpaired student’s t-test or one-way Anova analysis of variance with the Bonferroni post hoc test. P values <0.001, 0.01 and 0.05 were marked by three, two and one asterisks, respectively. P values >0.05 were considered not significant and marked by ns. The SEM for the 100% values are derived from variations between absolute values of independent experiments performed with the same batch of RFL-6 cells on the same day.

## Results

### Partial Dependency of nNOS Activity in Human A673 Neuroepithelioma Cells on Extracellular Arginine

**Figure 1:**
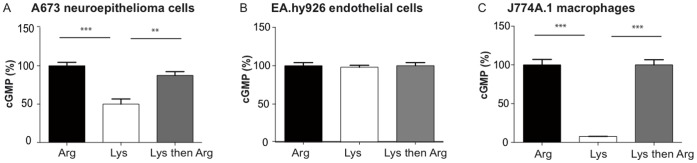
Individual NOS isoforms differ in their dependency on extracellular arginine. NO production was measured indirectly by cGMP formation in RFL-6 reporter cells exposed to supernatants of confluent human A673 neuroepithelioma (**A**), human EA.hy926 endothelial (**B**) or LPS-stimulated murine J774A.1 macrophage cells (**C**). Confluent NO-producing cells were pre-incubated for 2 hours in LS containing either 1 mM arginine (dark columns) or 1 mM lysine (white and grey columns) at 37°C. They were then incubated consecutively two times for 2 min each in LS containing the same amino acids and in the case of A673 and EA.hy926 cells also 10 µM calcium ionophore A23187. The cells represented in the grey columns were exposed to 1 mM lysine and 1 mM arginine, respectively, in the 1^st^ and 2^nd^ incubation. Supernatants of each incubation were singularly transferred to RFL-6 reporter cells, left for 2-minutes and then the cGMP content of the reporter cells was determined by radioimmunoassay. (Here, only values of the 2^nd^ incubations are shown). Values obtained were calculated as % of the mean of the values obtained from the corresponding control cells incubated in 1 mM arginine. The basal cGMP content of the RFL-6 cells was subtracted. Columns represent mean ± S.E.M. (n = 3–9, one way ANOVA with Bonferroni post hoc test).

**Figure 2:**
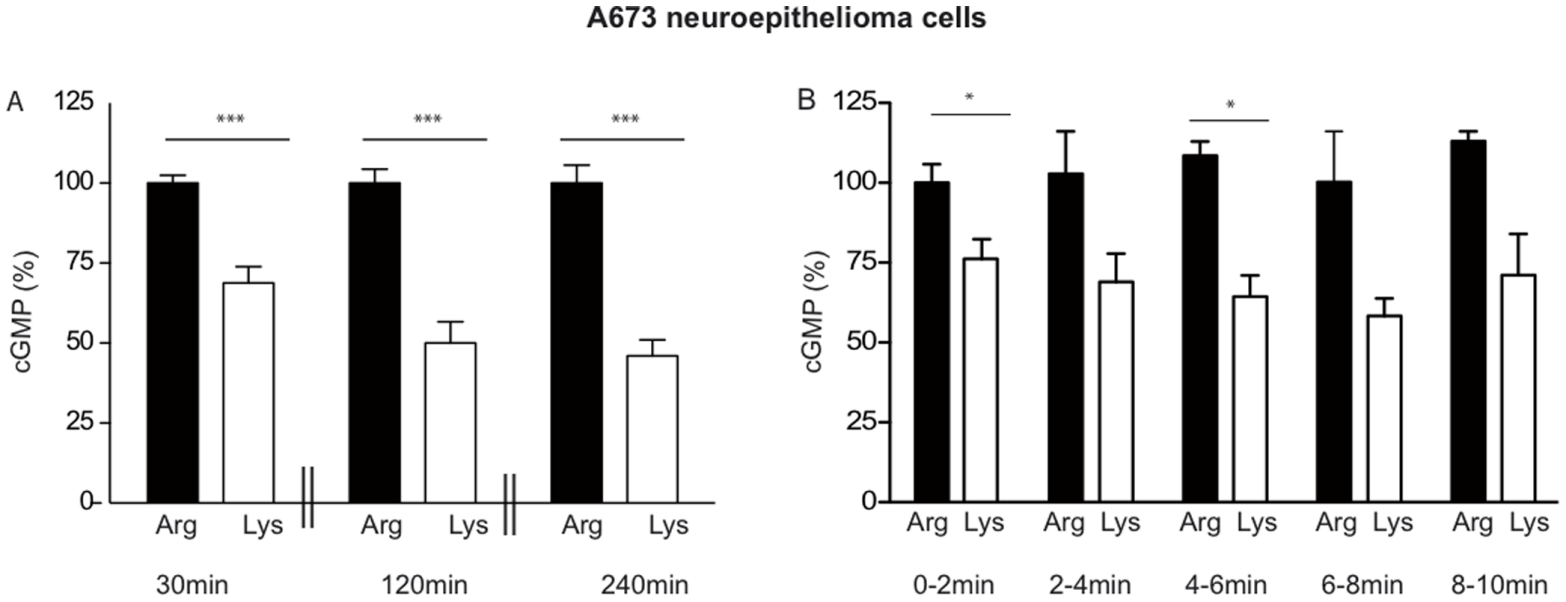
Preservation of residual nNOS activity also under prolonged arginine withdrawal in human A673 neuroepithelioma cells. (**A**) NO measurements were performed indirectly by cGMP formation in RFL-6 reporter cells as described in Figure 1(A), except that the pre-incubation in LS containing either 1 mM arginine (dark columns) or 1 mM lysine (white columns) was for 30, 120, or 240 min as indicated and only one transfer each was performed after stimulation with calcium ionophore. After substraction of the basal cGMP content of the RFL-6 cells, values obtained from cells incubated in 1 mM lysine were calculated as % of the mean of the values obtained from the corresponding control cells incubated in 1 mM arginine. Columns represent mean ± S.E.M. (n = 9–12, unpaired student’s t-test). (**B**) Cells were preincubated in LS containing either 1 mM arginine (dark columns) or 1 mM lysine (white columns) for 120 min. NO measurements and data analyses were performed as in Figure 1 (A), except that the same A673 cells were assayed five times consecutively. After each transfer, fresh LS supplemented with the indicated amino acid and 10 µM calcium ionophore A23187 was added to the A673 cells, incubated for 2 min and transferred to new RFL-6 cells. The basal cGMP content of the RFL-6 cells was subtracted. The values obtained from cells incubated in 1 mM lysine were calculated as % of the mean of the values obtained from the corresponding control cells incubated in 1 mM arginine for 0–2 min. Columns represent mean ± S.E.M. (n = 6, one way ANOVA with Bonferroni post-hoc test).

To find out if nNOS is either dependent (like iNOS) or not (like eNOS) on extracellular substrate supply via amino acid transporters, nNOS activity in human A673 neuroepithelioma cells was determined under conditions of ample arginine supply and arginine depletion, respectively, and directly compared to eNOS activity in EA.hy926 endothelial cells and iNOS activity in LPS-stimulated J774A.1 macrophages under the same conditions (Figure 1). Depletion of the arginine pool directly exchangeable with the extracellular space (arginine-pool I) was accomplished by a 2 h incubation in buffer containing the cationic amino acid lysine that is exchanged against intracellular arginine via cationic or system y^+^L amino acid transporters (CAT and y^+^LAT, respectively) (Figure S2). In A673 and EA.hy926 cells, NO production was stimulated by addition of calcium ionophore during the last 2 minutes of incubation. NO in cell supernatants was quantified via induction of cGMP formation in RFL-6 reporter cells. As observed for EA.hy926 endothelial cells, cGMP formation induced by supernatants of A673 and TGW-nu-I cells, was strongly stimulated when the NOS-expressing cells were incubated with the Ca^2+^ ionophore A23187 (thus activating the Ca^2+^-dependent nNOS) and abolished in the presence of the NOS inhibitor L-NAME or the NO scavenger 2-Phenyl-4,4,5,5-tetramethylimidazoline-1-oxyl 3-oxide (Figures S3A and S3B). This demonstrates that cGMP formation in the reporter cells mirrors in fact nNOS activity in the two cell lines. In A673 neuronal cells about 50% of nNOS activity was maintained during lysine-induced arginine depletion (Figure 1A). In contrast, NO-production in EA.hy926 and J774A.1 macrophage cells was completely independent of and dependent on extracellular arginine, respectively, (Figure 1B and C), as previously observed [34] [21]. In both, lysine-incubated A673 and J774A.1 cells, NOS activity was completely restored upon addition of extracellular arginine, demonstrating that the reduced NO production under arginine-depletion was merely due to substrate limitation (Figure 1, grey columns). When the pre-incubation time with lysine was extended from 30 min to up to 4 h (Figure 2A), a slightly stronger inhibition was observed (69% nNOS activity after 30 min versus 46% after 120 min). However, even after 4 h lysine incubation, nearly half of the nNOS activity (46% ±5) was still preserved. In addition, nNOS activity in lysine-preincubated cells (30 min), stimulated with Ca^2+^-ionophore in the continued presence of lysine was stable over five consecutive measurements: NO-production remained at about 70% of the maximal value obtained with arginine-incubated cells (Figure 2B). Consequently, there must be intracellular arginine sources for nNOS in A673 cells that can be used in addition to arginine pool I.

### A673 Cells cannot use Citrulline-derived Arginine for NO Synthesis

**Figure 3:**
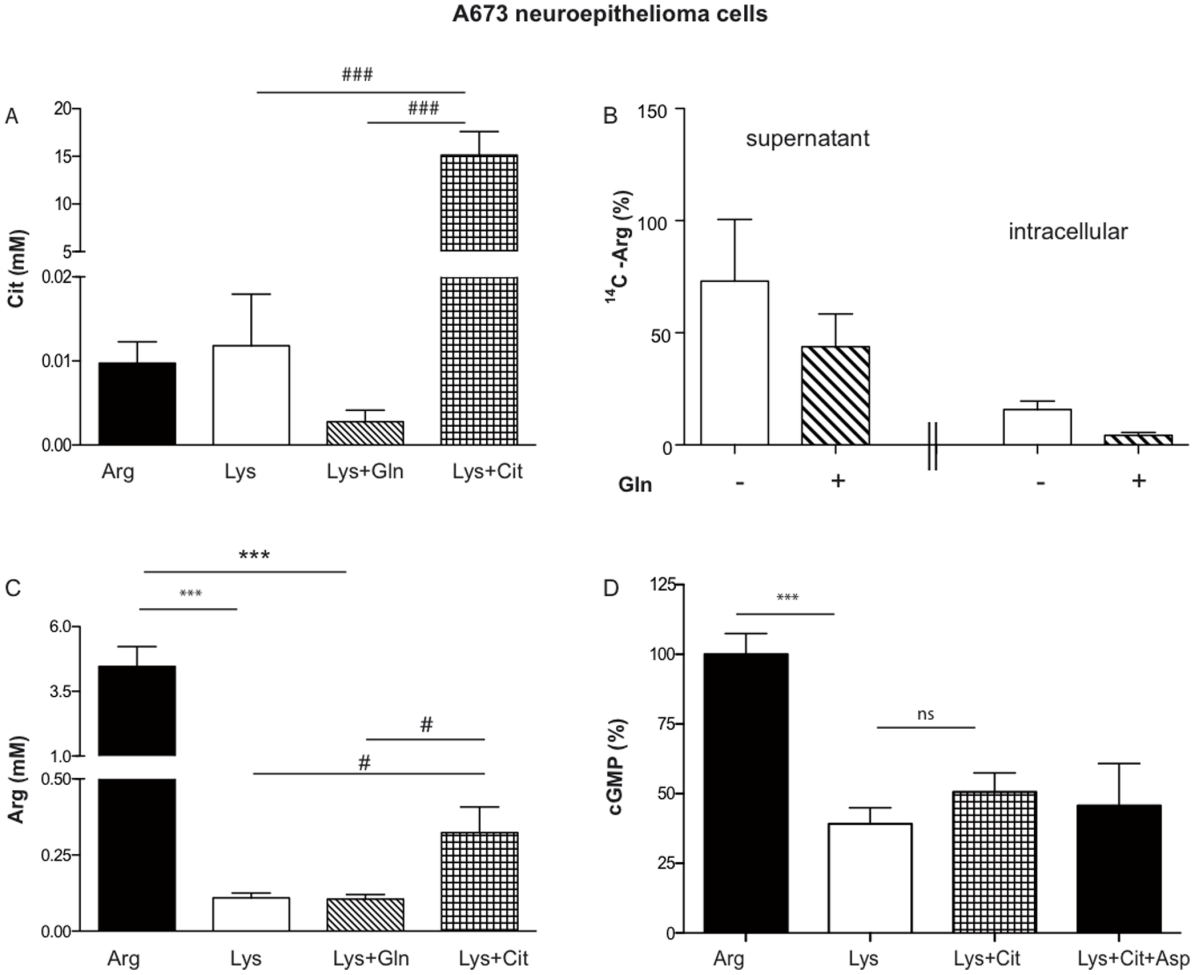
A673 neuroepithelioma cells take up extracellular citrulline and convert it to arginine, but do not use this arginine for NO-synthesis. (**A**) and (**C**): Confluent A673 cells were preincubated for 30 min in LS containing 1 mM of each indicated amino acid. The cells were then washed 3 times in ice-cold PBS and amino acids were extracted with 70% ethanol. Intracellular amino acid concentrations were calculated from the amino acid content of the cell lysates measured by HPLC, the cell volume of A673 cells (determined as 94±16 fL), and the cell number (mean ± S.E.M., n = 7–9, one way ANOVA with Bonferroni post hoc test). * significant difference to arginine-incubated cells, #: significant difference to cells incubated with lysine and citrulline (in C without consideration of the arginine-incubated cells). (**B**) A673 cells were incubated for 30 min at 37°C in LS supplemented with 1 mM lysine, 10.8 µM [^14^C]-citrulline, 100 µM L-NAME and with (dashed columns) or without (white columns) 1 mM glutamine as indicated. TLC analyses of supernatants and cell lysates were performed as detailed in the methods section. The [^14^C]-arginine spots were quantified using a phosphor-imager. All values were calculated as % of the mean of the values obtained in total from cells incubated without glutamine. Columns represent mean ± S.E.M. (n = 4, unpaired students t-test). (**D**) NO measurements and data analyses were performed as described in Figure 1. Thirty min preincubations and transfers were performed in LS with the indicated amino acids (single or in combination): 1 mM arginine, 1 mM lysine, 4 mM citrulline, 4 mM aspartic acid. Columns represent mean ± S.E.M. (n = 4–6, one way ANOVA with Bonferroni post hoc test).

To find out if A673 cells take up citrulline, intracellular concentrations of the amino acid were determined by HPLC in arginine-depleted cells supplemented or not with citrulline. Citrulline incubation raised the intracellular citrulline concentration from about 0.01 to 15 mM (Figure 3A), demonstrating that A673 cells are able to take up extracellular citrulline. Although cells incubated with glutamine and lysine exhibited lower intracellular citrulline concentrations than cells incubated in lysine alone, this difference was not significant (Figure 3A). Using ^14^C-labelled citrulline, we established that A673 neuronal cells were indeed able to convert citrulline to arginine, at a similar rate as EAhy926 endothelial cells (data not shown). Analyzing the ^14^C-arginine content in cells and their supernatants, we observed overall reduced levels of ^14^C-arginine in A673 cells incubated in glutamine and lysine compared to cells incubated in lysine alone (Figure 3B). Glutamine thus seems to reduce the citrulline to arginine conversion of A673 cells. To evaluate the contribution of the recycling pathway to overall intracellular arginine concentrations, HPLC determinations were performed in arginine-depleted cells supplemented or not with citrulline. Cells incubated with citrulline and lysine (1 mM each) exhibited a threefold higher intracellular arginine concentration compared to cells incubated with lysine alone or with lysine and glutamine (Figure 3C). In spite of efficient uptake of extracellular citrulline and recycling to arginine, an incubation with citrulline did not lead to a restoration of NO-production in arginine-depleted A673 cells (Figure 3D). An additional offer of aspartic acid, which is also necessary for citrulline to arginine recycling, did not increase NO production either (Figure 3D).

### Lysosomal and Proteasomal Protein Breakdown as Substrate Source for nNOS

**Figure 4:**
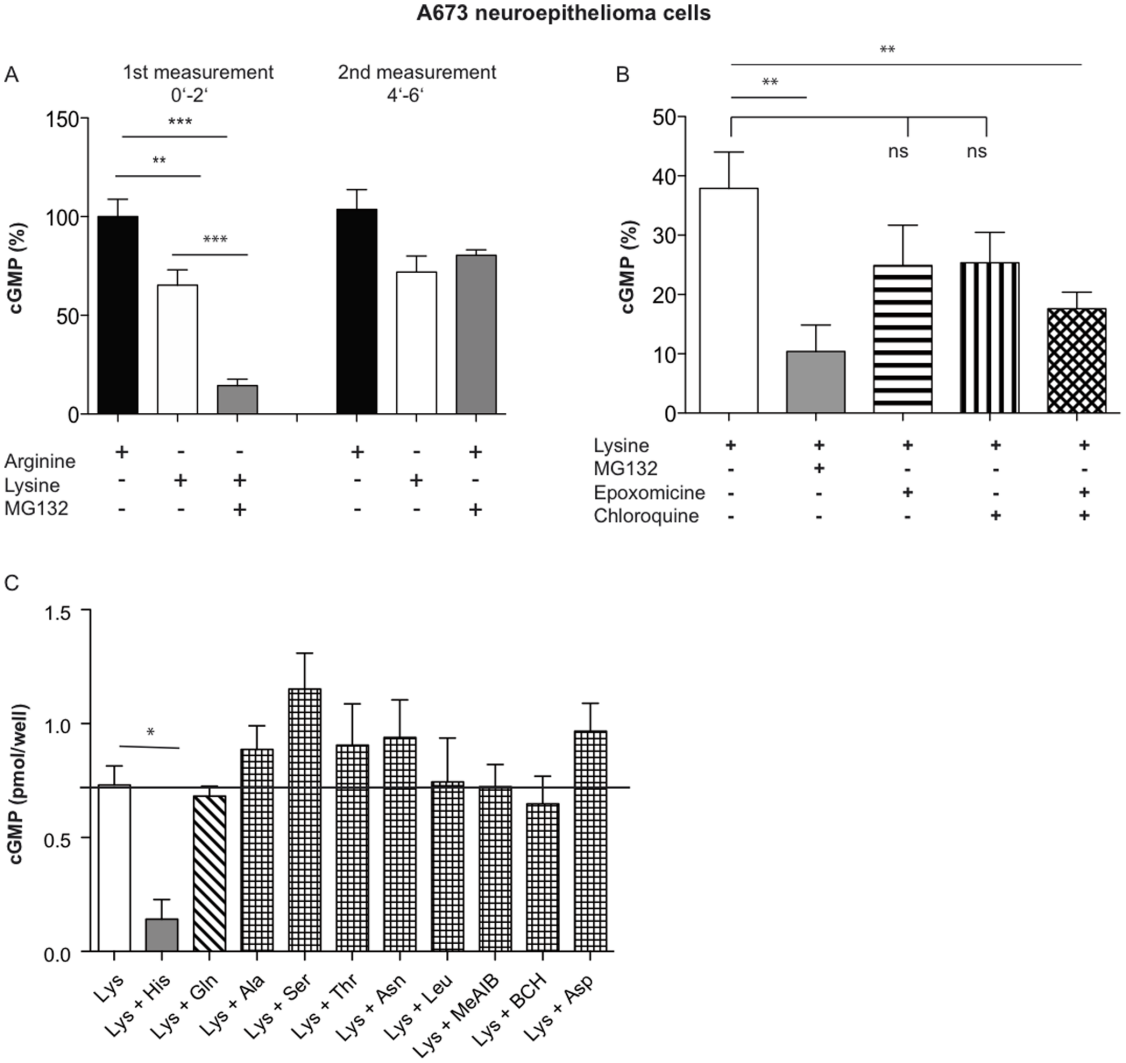
Lysosomal and proteasomal protein degradation serve as substrate source for nNOS. NO measurements and data analyses were performed as described in Figure 1 by RFL-6 reporter cell assays, this time in 24 well plates. (**A**) Thirty min pre-incubations and transfers (0–2′) were performed in LS supplemented with 1 mM arginine, 1 mM lysine or 1 mM lysine plus 1 µM of the inhibitor of proteasomal and lysosomal protein breakdown MG132 as indicated (1^st^ measurement). After the transfer, new LS containing either the same supplements as in the first incubation (black and white columns) or MG132 and 1 mM arginine (gray column), was added to the same A673 cells. After 2 min, each buffer was renewed and after further 2 min, a second transfer was performed (4–6′, 2^nd^ measurement). The basal cGMP content of the RFL-6 cells was subtracted. Columns represent mean ± S.E.M. (n = 6, one way ANOVA with Bonferroni post hoc test). (**B**) Thirty min pre-incubations and transfers were performed in LS supplemented with 1 mM arginine, 1 mM lysine or 1 mM lysine plus 100 µM chloroquine or/and 5 µM epoxomicin as indicated. The basal cGMP content of the RFL-6 cells was subtracted. Columns represent mean ± S.E.M. (n = 8, one way ANOVA with Bonferroni post hoc test). (**C**) Thirty min preincubations and transfers were performed in LS containing 1 mM arginine or 1 mM lysine either alone or in combination with histidine, glutamine, alanine, serine, threonine, asparagine, leucine, aspartic acid, 2-(methylamino)-isobutyric acid (MeAIB), or 2-aminobicyclo-(2,2,1)-heptane-2-carboxylic acid (BCH), (4 mM each). The basal cGMP content of the RFL-6 cells was subtracted. Columns represent mean ± S.E.M. (n = 3, one way ANOVA with Bonferroni post hoc test).

**Figure 5:**
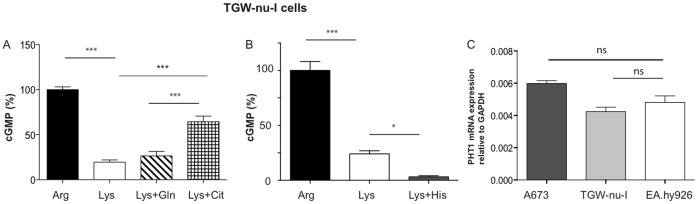
nNOS in TGW-nu-I cells can use both, citrulline-to-arginine recycling and protein degradation as intracellular substrate source. (**A**) and (**B**) NO measurements and data analyses were performed by RFL-6 reporter cell assays as described in Figure 1. Thirty min pre-incubations and transfers were performed in LS with the indicated amino acids (single or in combination): 1 mM arginine, 1 mM lysine, 4 mM glutamine, 4 mM citrulline, or 4 mM histidine. The basal cGMP content of the RFL-6 cells was subtracted. The values obtained from cells incubated in 1mM lysine were calculated as % of the mean of the values obtained from the corresponding control cells incubated in 1 mM arginine. Columns represent mean ± S.E.M. **(**n = 6 (A) and n = 6–12 (B), one way ANOVA with Bonferroni post hoc test). (**C**) Total RNA from A673, TGW-nu-I, and EA.hy926 cells (0.5 µg both) was analyzed by qRT/PCR for PHT1 expression as described in the methods section. GAPDH was chosen as housekeeping gene for relative determinations. (means ± S.E.M., n = 3).

The inhibitor of proteasomal and lysosomal protein breakdown MG132 [35], applied in a concentration of 1 µM, reduced the nNOS activity of lysine-incubated A673 cells further from about 65% to 14% of arginine-incubated cells, respectively (Figure 4A, first measurement). NO production was completely restored upon addition of arginine in the continuous presence of the inhibitor (Figure 4A, second measurement), demonstrating that the inhibitory effect was exclusively due to reduction in substrate supply. To quantify the contribution of each protein degradation pathway, we used specific inhibitors of lysosomal (chloroquine 100 µM) and proteasomal (epoxomycin 5 µM) protein degradation (Figure 4B). There was no significant reduction of nNOS activity by either compound, although a trend for a slight reduction could be noticed. A significant reduction in NOS activity was only observed when the two inhibitors were combined. In endothelial cells, we have observed that histidine interferes with eNOS activity most likely by inducing an exchange of intracellular peptides via the peptide histidine transporter PHT1. Indeed, histidine reduced also nNOS activity in lysine-incubated A673 cells and to a similar extent as MG132 (Figure 4C). In contrast, none of the other neutral amino acids (including glutamine) exhibited an inhibitory effect on nNOS activity. The histidine effect can thus not be explained by extraction of intracellular arginine via y^+^L transporters. Quantitative RT/PCR revealed that hPHT1 mRNA was expressed in A673 cells at a similar level as in EA.hy926 cells (Figure 5C) [22]. Only a very weak expression of the other peptide-transporters PEPT1 and 2 was found in A673 cells (data not shown).

### nNOS Activity in TGW-nu-I Neuroblastoma Cells

Substrate supply of nNOS in TGW-nu-I neuroblastoma cells was similar to A673 cells. However, lysine incubation reduced nNOS activity to a greater extent than in A673 cells, indicating that protein degradation is a less important arginine source in these cells (Figure 5A). Histidine completely abolished NO production in lysine-incubated cells (Figure 5B), probably also via exchange against intracellular peptides mediated by PHT1 that was expressed in TGW-nu cells at a similar level as in EA.hy926 cells (Figure 5C). In contrast to A673 cells, extracellular citrulline increased NO production in TGW-nu-I cells under lysine-depletion, demonstrating that the recycling pathway can be used as substrate source for nNOS in these cells (Figure 5A). However, glutamine did not further reduce NO production in lysine-incubated cells. This indicates that arginine generated from endogenous citrulline did not serve as nNOS substrate.

## Discussion

**Figure 6:**
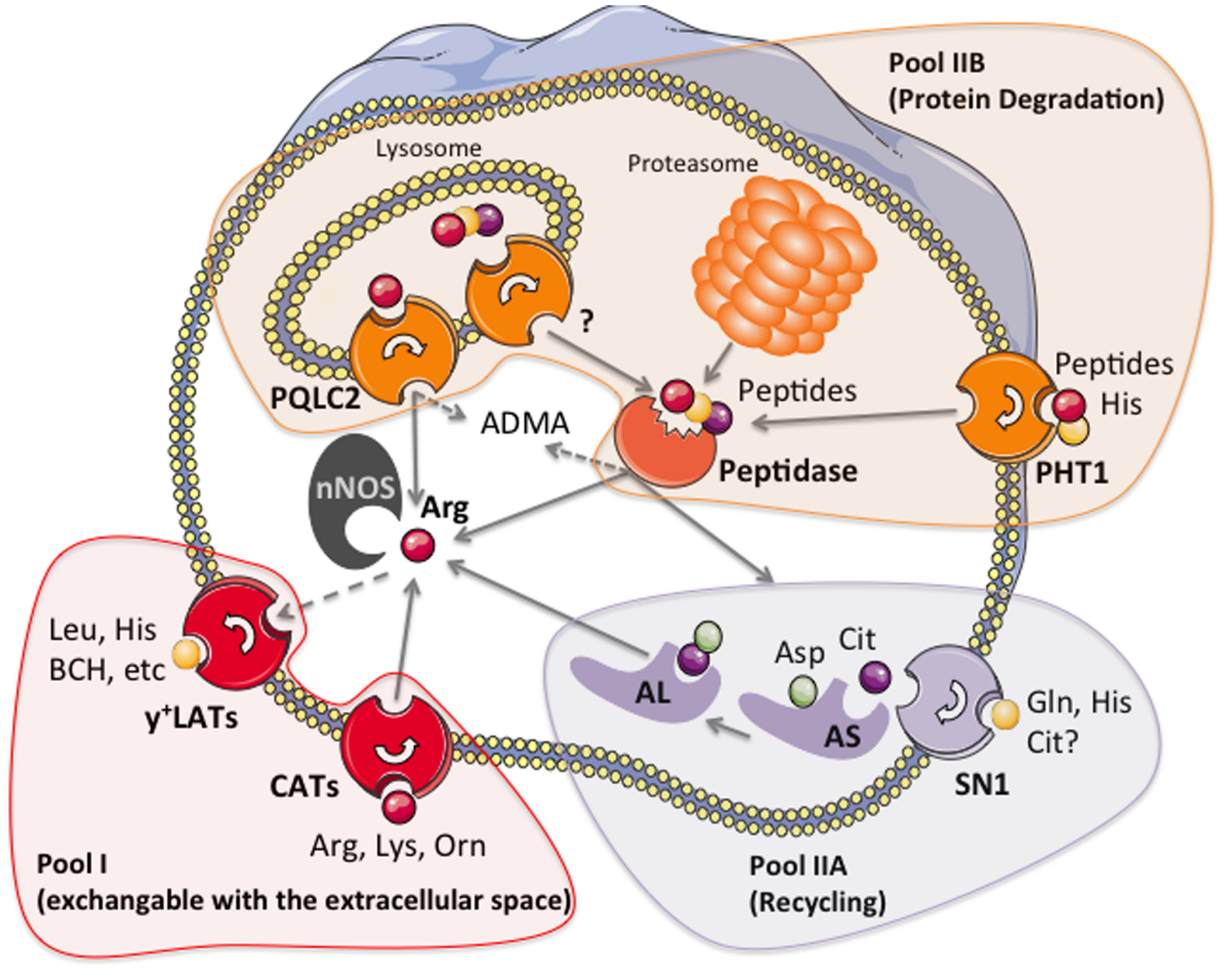
Scheme of the different arginine pools for nNOS in A673 and TGW-nu-I neuronal cells. The scheme depicts different arginine pools in neuronal cells: The exchangeable pool I (red) consists of plasma membrane transporters that take up arginine from the extracellular space (CATs, for cationic amino acid transporter), but can also extract arginine from cells in exchange against other cationic (CATs and y+LATs) or neutral amino acids (y+LATs). Pool IIA (purple) is made up of the so-called recycling enzymes argininosuccinate synthase and lyase that convert citrulline to arginine. This pool is only available to nNOS in TGW-nu-I cells supplemented with extracellular citrulline. A673 cells can neither use endogenous nor exogenous citrulline as source for nNOS substrate. Pool IIB (orange) is present in both cell types. It comprises proteasomal and lysosomal protein degradation. Free arginine or arginine-containing peptides generated by the latter exit the lysosome, respectively, by the newly discovered amino acid transporter PQLC2 and peptide transporters yet to be identified. Also, the peptidase(s) that sets arginine free from peptides have not been specified to date. Peptides may also enter (or exit) neuronal cells by peptide transporters of the plasma membrane, with PHT1 exhibiting the most pronounced expression in both, A673 and TGW-nu-I cells. Pool IIB also generates the NOS inhibitor asymmetric dimethyl arginine (ADMA).

In this study, we investigated the different arginine pools that can be used as substrate source for human nNOS in two different cell types, A673 neuroepithelioma and TGW-nu-I neuroblastoma cells (see scheme in Figure 6). In both cell types, nNOS was only partially dependent on arginine supply from pool I that is freely exchangeable between the intra- and extracellular space via plasma membrane transporters: depletion of that pool by incubation of the cells in high concentrations of extracellular lysine (that serves as a electro-neutral exchange substrate for arginine) led only to a partial reduction in nNOS activity, in spite of a dramatic reduction in intracellular arginine concentrations (Figure 3C).

Substrate supply to nNOS in these cells is thus intermediate between eNOS in endothelial cells (no dependence on pool I) and iNOS in macrophages (complete dependence on pool I). Our previous studies as well as a side by side comparison in the present study establish these differences [34] [21].

Interestingly in A673 cells, the drop in intracellular arginine concentrations upon a 30 min lysine incubation was similar as previously observed in macrophages and endothelial cells [34] [21] – (Figure S2). The dependency of the macrophage effector mechanism, later identified as iNOS-derived NO, on extracellular arginine has been observed even before the discovery of NO and iNOS and has since been found in numerous studies [36]. Sustained NO synthesis by iNOS in murine macrophages requires even the induction of a specific cationic amino acid transporter, CAT-2B, underlining the importance of arginine pool I for iNOS in these cells [37] [38]. nNOS investigated in the present study was clearly less dependent on this substrate pool.

As in EA.hy926 cells, the predominant arginine importer in A623 cells seems to be CAT-1 [39]. A673 cells express additionally but weakly CAT-2B and -3. Both cell types exhibit also considerable expression of y^+^LAT arginine exporters. CAT-1, CAT-2B and y^+^LAT1 are also expressed in activated J774A1 macrophages [34] [40]. Hence, differences in the substrate supply to NOS do not seem to be due to differences in plasma membrane transporters in the different cell types.

The capacity of the intracellular substrate sources (pool II) in nNOS-expressing cells seems however to vary as lysine incubation did not inhibit nNOS to the same extent in all experiments. However, the capacity in a given experiment seemed to be pretty stable upon at least 5 repeated measurements during constant Ca^2+^ stimulation as well as upon lysine preincubation over up to four hours. As protein degradation (substrate pool IIB) made up by far the largest part of the intracellular substrate source, the variation is probably a function of the overall capacity of the cells to degrade proteins for replenishments of amino acid pools. Both, lysosomes and proteasomes seem to restock the nNOS substrate pool IIB, when the exchangeable arginine pool I is depleted by lysine incubation. Only the simultaneous blockage of both protein degradation pathways either by MG132 or the combination of chloroquine and epoxomycin resulted in a significant NOS inhibition while individual inhibition of either lysosomes and proteasomes had no effect. This indicates that each source alone can fully sustain nNOS substrate pool IIB. Unless each protein degradation pathway is induced by the inhibition of the respective other pathway (and thus provides exactly the substrate part missing from the inhibited source), pool IIB must be limited by factors other than provision of peptides from protein breakdown. This phenomenon has already been observed in lysine-incubated endothelial cells, where protein breakdown delivers about 80% of eNOS substrate, whilst from results of individual inhibition of either pathways one would anticipate that lysosomes and proteasomes together should provide enough arginine to maintain full NO synthesis [22].

A toxic effect of the simultaneous inhibition of both pathways on either NOS isoform can be excluded as NO synthesis could always be completely restored by supplementing L-arginine in the extracellular buffer in the continued presence of the protease inhibitors (Fig. 4A, right side). Other than in endothelial cells, we did not observe a predominant contribution of lysosomal protein degradation to replenishment of pool IIB in A673 cells. Putative downstream events that may limit the capacity of pool IIB are discussed below.

Our study further demonstrates that nNOS in both, A673 neuroepithelioma and TGW-nu-I neuroblastoma cells can fast and efficiently be nourished by extracellular arginine (arginine pool I that is freely exchangeable between the intra- and extracellular space): Under all experimental conditions (e.g. after arginine depletion with extracellular lysine and inhibition of protein breakdown), NO synthesis fully recovered within 4 min upon addition of extracellular arginine.

Pool I should carry ample substrate under normal conditions: An arginine concentration of approximately 30 µM has been measured in cerebral spinal fluid in humans [41]. At a membrane potential of -60 mV, this should result in an about tenfold higher intracellular arginine concentration largely sufficient to saturate nNOS (K_M_: 2 µM) [42]. However, under conditions of extensive arginine consumption, e.g. when arginase activity is high, local arginine concentrations may drop considerably. Arginase does not only degrade arginine but also produces ornithine, another cationic amino acid, that is - like lysine - exchanged against arginine via cationic amino acid transporters. This assures continuous feeding of arginine to arginase while neighboring cells are continuously depleted from arginine via exchange against ornithine [43]. Our experiments - where we used lysine incubation to mimic this situation - suggest that in the presence of arginase-producing cells in the surrounding, nNOS would continue to produce NO at about half maximal rate, while iNOS would be shut off. It remains to be explored however, under which conditions and for how long protein degradation is able to sustain NO synthesis in primary nNOS-expressing cells in vivo that may behave differently from the cultured cells investigated. Asymmetric dimethyl-L-arginine (ADMA), the most abundant endogenous NOS inhibitor originates also from protein breakdown [44]. Arginine methylation in the degraded proteins may thus limit the capacity of pool IIB as a substrate source of nNOS.

In contrast to our finding of only a 45–70% contribution of arginine pool I to nNOS supply, Bae and collegues found nNOS activity in murine tyrosine hydroxylase positive CAD cells to be almost completely dependent on extracellular arginine concentrations up to 300µM, weigh above the K_M_ of nNOS [23]. CAD cells lack argininosuccinate synthase, the rate-limiting enzyme for the recycling of citrulline to arginine, leaving protein breakdown the only possible intracellular arginine source. In this study, NOS activity was measured over five hours and thus arginine consumption by other pathways during this long period may have contributed to the arginine dependence. CAD cells express also arginase I and II that compete with nNOS for arginine (see above). Therefore, with arginase present within the nNOS-expressing cell, intracellular arginine supply by protein breakdown may not suffice to sustain NO synthesis.

One may ask why the arginine liberated by either lysosmal or proteasomal protein degradation was not (entirely) exchanged against extracellular lysine in our experiments. Interestingly, although A673 cells express 4F2hc/y^+^LATs [39] that mediate a Na^+^-dependent active export of arginine (and other cationic amino acids) against extracellular neutral amino acids (such as leucine), neither leucine nor other 4F2hc/y^+^LAT substrates were able to reduce nNOS activity in A673 cells further than lysine alone. This indicates a strong coupling of arginine generation from protein breakdown with nNOS, similar to that observed for eNOS in endothelial cells. Both, lysosomes and proteasomes generate peptides that are further broken down to amino acids by peptidases (Figure S2). A close proximity of nNOS to such peptidase(s) could explain the efficient substrate supply in spite of high concentrations of extracellular exchange substrate. The limitation of the capacity of pool IIB discussed above may thus also be explained by a rate limiting activity of these peptidase(s).

A further rate-limiting step in the delivery of substrate to nNOS may be the transport of lysosome-derived arginine or arginine-containing peptides to the cytosol (Figure S2). A lysosomal transporter for cationic amino acid has recently been discovered by two independent groups [45] [46]. It remains to be determined if it plays an important role in the substrate supply to nNOS. In contrast, lysosomal peptide transporters are not well characterized. The peptide/histidine transporter PHT2 has been reported to localize to lysosomes [47]. However, in the cells investigated in the present study, PHT1, so far only detected in the plasma membrane [48], was the predominant peptide transporter expressed.

Neuronal expression of PHT1 is consistent with a strong expression of PHT1 in rat brain [48]. If this transporter is responsible for the pronounced nNOS inhibition induced by histidine remains to be determined. As already discussed for endothelial cells, this inhibition may be explained by a PHT1-mediated export of intracellular arginine-containing peptides in exchange with extracellular histidine. However, such an exchange function has not yet been described for PHT1. Interestingly, a peptide transporter (TAPL) that mediates concentrative uptake of a broad range of different peptides into lysosomes is strongly induced in macrophages upon maturation [49]. It is tempting to speculate that this transporter reduces the availability of peptides in the cytoplasm of iNOS-expressing macrophages and thus contributes to the dependence of these cells on extracellular arginine for NO synthesis.

Under lysine incubation, arginine generated from extracellularly-derived citrulline was only available to nNOS in TGW-nu-I neuroblastoma, but not in A673 neuroepithelioma cells. This is in spite of the fact that A673 cells exhibited similar citrulline uptake and conversion to arginine as EA.hy926 endothelial cells where extracellularly-offered citrulline can fully sustain NO-synthesis. As glutamine did not reduce nNOS activity in either TGW-nu-I neuroblastoma or A673 neuroepithelioma, endogenous citrulline does not seem to contribute to the supply of nNOS substrate in these cells, whereas 25% of NOS-substrate in endothelial cells is supplied by the recycling from endogenous citrulline to arginine. In general, citrulline seems to be a better substrate source for eNOS than for nNOS in the cells investigated. The difference in substrate supply seems to be due to differences in coupling of the arginine source to the NOS rather than arginine production itself. Cell type-dependent usage of intracellular arginine sources has also been found for eNOS: When expressed exogenously in ECV304 bladder carcinoma cells, eNOS is almost completely dependent on extracellular arginine [21]. Also, a number of studies show an influence of arginine transport on eNOS activity under pathophysiological conditions [50].

In contrast to the observation that the arginine pool for eNOS and nNOS cannot be (entirely) depleted by extracellular exchange substrate, our previous experiments with endothelial cells demonstrated, that a large part of L-arginine derived from protein breakdown is found in the extracellular space. This indicates that most, if not all, L-arginine generated in the cytoplasm is exchangeable with the extracellular space. Preservation of substrate supply to NOS under L-lysine incubation could thus be simply explained by a gradient between the L-arginine-producing source and the extracellular space sufficient to saturate the high affinity NOS isoforms. In this case, differences between eNOS and nNOS, but also between different cell types could be due to a different subcellular localization of the respective NOS isoform in the respective cell type relative to the L-arginine producing system. Interestingly, nNOS contains a PDZ domain that determines not only its subcellular distribution and interaction with other (PDZ-containing) proteins, but also its enzyme activity [51].

Alternatively, a small part of the L-arginine generated by proteasomes and lysosomes may not be exchangeable with the extracellular space, but directly delivered to NOS by a yet unidentified peptidase or lysosomal transporter for cationic amino acids, respectively (Figure S2). As discussed above, such coupling or close proximity between peptidase and nNOS could explain why substrate contribution from protein degradation in A673 cells was capped to an average of 50% irrespective of the availability of only lysosomal or proteasomal or both protein degradation pathways.

Similarly, the arginine produced by argininosuccinate synthase and argininosuccinate lyase from citrulline could either be handed over directly to NOS or produced in its vicinity thus enabling substrate supply even under lysine-induced arginine depletion. Differences in the behavior between nNOS and eNOS in different cell types could again be explained by differences in the subcellular localization of the arginine producing and consuming system relative to each other. In contrast to ECV cells, that use neither protein breakdown nor recycling for eNOS supply, the two cell types investigated in our present study, exhibited an efficient usage of the first, but not the second intracellular substrate source for nNOS. Accordingly, we found nNOS to be inhibited by histidine (supposed to interfere with arginine supply from protein breakdown), but not glutamine (supposed to interfere with citrulline to arginine recycling), while both amino acids inhibit eNOS in endothelial cells (although to a different degree). This supports the notion that the two amino acids may act via different pathways to inhibit NOS.

In summary, our data indicate that protein degradation (but not arginine generation from citrulline) is a stable substrate source for nNOS in neuronal cells leaving the enzyme activity at about half maximal rate when the freely exchangeable arginine pool is depleted. Arginine depletion may thus represent a promising approach to master the tightrope walk between preserving beneficial and limiting detrimental NO synthesis in the brain.

On the basis of our cell culture experiments it would be interesting to investigate the specific contribution of each possible arginine pool to the substrate supply of nNOS in neuronal cells in different areas of the human brain and under certain illnesses. Gaining a further understanding of this complex interplay would lead to a better comprehension of neuronal diseases involving excessive NO production.

### Conclusions

To evaluate if nNOS activity can be controlled by limiting its substrate supply, we characterized the arginine sources for nNOS in A673 neuroepithelioma and TGW-nu-I neuroblastoma cells.

We found in both cell types that.

nNOS can be fast and efficiently nourished by extracellular arginine that enters the cells via membrane transporters.
intracellular arginine sources partially sustain NO synthesis with protein breakdown making up by far the largest part of that substrate source.
a combination of lysine and histidine inhibits NO synthesis efficiently.

Citrulline-derived arginine sustained NO synthesis only in TGW-nu-I, but not A673 cells.

Our data demonstrate that nNOS activity can be modulated by substrate availability. However further research is necessary to evaluate the substrate supply of primary nNOS expressing cells in the central nervous system.

## Supporting Information

Figure S1 **Scheme of different arginine pools for eNOS in EA.hy.926 endothelial cells.** The scheme depicts different arginine pools in endothelial cells defined in our previous work [21], [22]: The exchangeable pool I (red) consists of plasma membrane transporters that take up arginine from the extracellular space (CATs, for cationic amino acid transporter), but can also extract arginine from cells in exchange against other cationic (CATs and y+LATs) or neutral amino acids (y+LATs). Pool IIA (purple) is made up of the so-called recycling enzymes argininosuccinate synthase and lyase that convert citrulline to arginine. In the presence of extracellular lysine, arginine synthesis from endogenous citrulline makes up about 25% of the eNOS substrate supply. When extracellular citrulline is supplemented, pool IIA can sustain NO synthesis to 100%. Pool IIB (orange) comprises proteasomal and lysosomal protein degradation and make up about 75% of eNOS substrate supply when the exchangeable pool I is depleted. Free arginine or arginine-containing peptides generated by the latter exit the lysosome, respectively, by the newly discovered amino acid transporter PQLC2 and peptide transporters yet to be identified. Also, the peptidase(s) that sets arginine free from peptides have not been specified to date. Peptides may also enter (or exit) endothelial cells by peptide transporters of the plasma membrane, with PHT1 exhibiting the most pronounced expression in EA.hy926 cells. Pool IIB also generates the NOS inhibitor asymmetric dimethyl arginine (ADMA).
(PDF)

Figure S2 **Comparison of intracellular arginine levels in EA.hy926, J774A.1 and A673 cells under extracellular arginine and lysine incubation.** The Figure compares intracellular arginine concentrations in the indicated cell lines after a 30 min incubation in either 1 mM L-arginine or 1 mM L-lysine. Date are derived from Figure 3c (A673) or from our previous work (EAhy.926 cells: Figure 7 [21], J774A1 cells: Figure 5B [34]).
(PDF)

Figure S3 **Induction of cGMP formation in RFL-6 reporter cells by supernatants of nNOS-expressing cells is highly stimulated by exposure of the latter to Ca2+-ionophore and abolished by the NOS inhibitor L-NAME and the NO scavenger PTIO.** Confluent A673 and TGW-nu-I cells grown in six well plates, were washed twice in LS and pre-incubated at 37°C for 30 min in LS containing 40 U/ml SOD and either 1 mM arginine (dark columns) or no amino acids (grey columns) as well as, where indicated, 0.1mM Nω-Nitro-L-arginine methyl ester (L-NAME) or 0.1mM 2-Phenyl-4,4,5,5-tetramethylimidazoline-1-oxyl 3-oxide (PTIO). The cells were then incubated for 2 min in the same LS, respectively, containing in addition 0.3 mM IBMX and where indicated 10 µM calcium-ionophore A23187. As described in Figure 1, supernatants were singularly transferred to RFL-6 reporter cells and left for another 2 min. The cGMP content of the RFL-6 cells was determined by radioimmunoassay. The basal cGMP content of the RFL-6 cells was subtracted. Columns represent mean ± S.E.M. (n = 3–6). Note that 100 µM L-NAME inhibited nNOS in TGW-nu-I cells only partly, when the cells were incubated in 1 mM L-arginine, most likely because of a insufficiently high inhibitor:substrate ratio. Accordingly, in the absence of exogenous substrate, L-NAME inhibited nNOS almost completely.
(PDF)
