# Supplementary figures and images for: Decoding the Substrate Supply to Human Neuronal Nitric Oxide Synthase
Source: PLoS One. 2013 Jul 9;8(7):e67707. doi: 10.1371/journal.pone.0067707 (PMC3706577; doi:10.1371/journal.pone.0067707)

Figure S1:

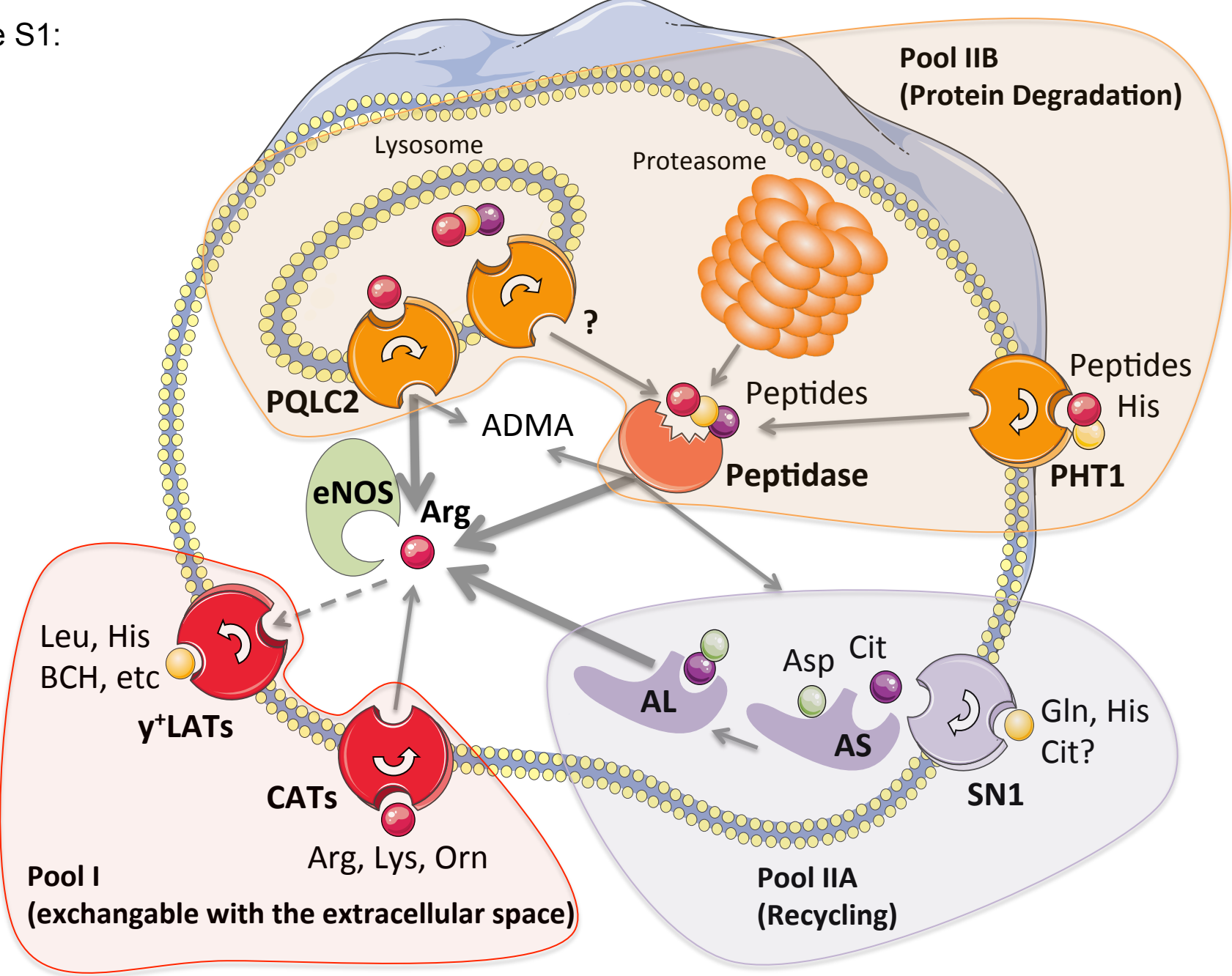

Supplement: Figure S1 — Scheme of different arginine pools for eNOS in EA.hy.926 endothelial cells. The scheme depicts different arginine pools in endothelial cells defined in our previous work [21], [22]: The exchangeable pool I (red) consists of plasma membrane transporters that take up arginine from the extracellular space (CATs, for cationic amino acid transporter), but can also extract arginine from cells in exchange against other cationic (CATs and y+LATs) or neutral amino acids (y+LATs). Pool IIA (purple) is made up of the so-called recycling enzymes argininosuccinate synthase and lyase that convert citrulline to arginine. In the presence of extracellular lysine, arginine synthesis from endogenous citrulline makes up about 25% of the eNOS substrate supply. When extracellular citrulline is supplemented, pool IIA can sustain NO synthesis to 100%. Pool IIB (orange) comprises proteasomal and lysosomal protein degradation and make up about 75% of eNOS substrate supply when the exchangeable pool I is depleted. Free arginine or arginine-containing peptides generated by the latter exit the lysosome, respectively, by the newly discovered amino acid transporter PQLC2 and peptide transporters yet to be identified. Also, the peptidase(s) that sets arginine free from peptides have not been specified to date. Peptides may also enter (or exit) endothelial cells by peptide transporters of the plasma membrane, with PHT1 exhibiting the most pronounced expression in EA.hy926 cells. Pool IIB also generates the NOS inhibitor asymmetric dimethyl arginine (ADMA). (PDF) [file pone.0067707.s001.pdf]

Figure S2:

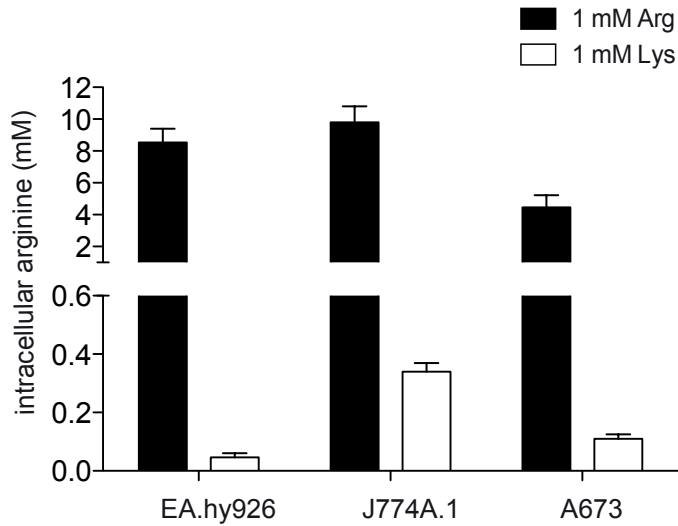

Supplement: Figure S2 — Comparison of intracellular arginine levels in EA.hy926, J774A.1 and A673 cells under extracellular arginine and lysine incubation. The Figure compares intracellular arginine concentrations in the indicated cell lines after a 30 min incubation in either 1 mM L-arginine or 1 mM L-lysine. Date are derived from Figure 3c (A673) or from our previous work (EAhy.926 cells: Figure 7 [21], J774A1 cells: Figure 5B [34]). (PDF) [file pone.0067707.s002.pdf]

Figure S3:

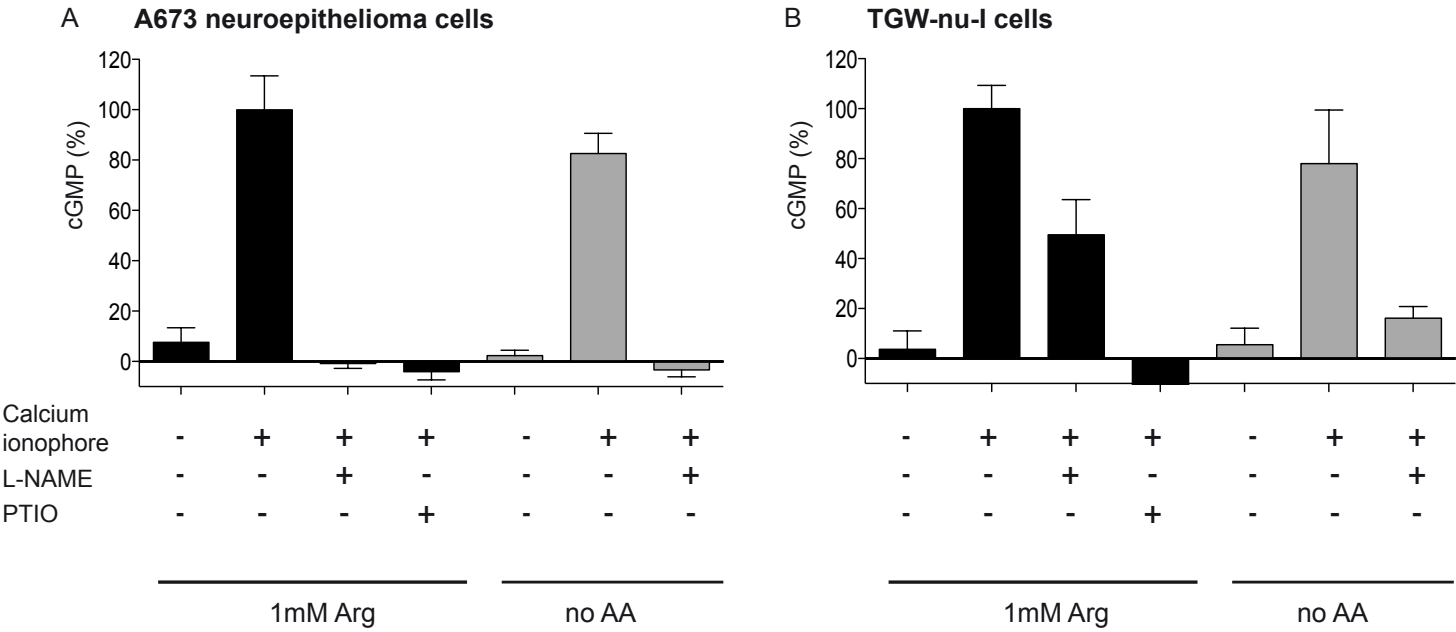

Supplement: Figure S3 — Induction of cGMP formation in RFL-6 reporter cells by supernatants of nNOS-expressing cells is highly stimulated by exposure of the latter to Ca2+-ionophore and abolished by the NOS inhibitor L-NAME and the NO scavenger PTIO. Confluent A673 and TGW-nu-I cells grown in six well plates, were washed twice in LS and pre-incubated at 37°C for 30 min in LS containing 40 U/ml SOD and either 1 mM arginine (dark columns) or no amino acids (grey columns) as well as, where indicated, 0.1mM Nω-Nitro-L-arginine methyl ester (L-NAME) or 0.1mM 2-Phenyl-4,4,5,5-tetramethylimidazoline-1-oxyl 3-oxide (PTIO). The cells were then incubated for 2 min in the same LS, respectively, containing in addition 0.3 mM IBMX and where indicated 10 µM calcium-ionophore A23187. As described in Figure 1, supernatants were singularly transferred to RFL-6 reporter cells and left for another 2 min. The cGMP content of the RFL-6 cells was determined by radioimmunoassay. The basal cGMP content of the RFL-6 cells was subtracted. Columns represent mean ± S.E.M. (n = 3–6). Note that 100 µM L-NAME inhibited nNOS in TGW-nu-I cells only partly, when the cells were incubated in 1 mM L-arginine, most likely because of a insufficiently high inhibitor:substrate ratio. Accordingly, in the absence of exogenous substrate, L-NAME inhibited nNOS almost completely. (PDF) [file pone.0067707.s003.pdf]
